# Association between adverse childhood experiences and type 2 diabetes mellitus in later life: A case-control study

**DOI:** 10.1371/journal.pgph.0002715

**Published:** 2024-06-25

**Authors:** Nilima Barman, Abul B. M. M. K. Islam, M. Atiqul Haque

**Affiliations:** 1 Department of Genetic Engineering and Biotechnology, University of Dhaka, Dhaka, Bangladesh; 2 Bangladesh Institute of Research and Rehabilitation of Diabetes, Endocrine and Metabolic Disorder (BIRDEM) General Hospital, Dhaka, Bangladesh; 3 Department of Public Health and Informatics, Bangabandhu Sheikh Mujib Medical University, Dhaka, Bangladesh; African Population and Health Research Center, KENYA

## Abstract

Adverse childhood experiences (ACEs) are potentially traumatic events that occur before 18 years of age. Studies emphasize the importance of childhood adversity as a risk factor for developing non-communicable diseases, including type-2 diabetes mellitus (T2DM) in adulthood. This case-control study involved 137 patients with T2DM and 134 non-diabetic adults of both genders (mean age 46.9 and 45.7 years, respectively). In addition to collecting socio-demographic, behavioral, and anthropological data, a 10-item ACE scale was utilized to gather information regarding childhood adversities, while perceived stress was assessed using the perceived stress scale-4. Fasting and 2-hour post glucose load blood sugar levels, HbA1c, and fasting lipid profiles were measured. Both univariable and multivariable binary logistic regression analyses were performed to investigate whether ACE is a potential risk factor for T2DM, with a significance level of 0.05. Around two-thirds of T2DM patients reported having ACE scores of 4 or higher, with the mean ACE score significantly higher in the case group than in the control group (3.96 vs. 3.34; p<0.0001). The logistic regression analysis found that T2DM was linked to female gender, hypertension, dyslipidemia, family history of DM, higher perceived stress, and a higher ACE score of 4 and above. After adjusting for confounding factors, individuals with an ACE score of 4 or higher had a significantly greater risk of developing T2DM (OR: 2.24; 95% CI 1.238–4.061). The study revealed a significant association between higher ACE scores and an increased risk of developing T2DM. As a recommendation, further investigation into the epigenetic mechanisms underlying this relationship is warranted.

## Introduction

As diabetes is estimated to affect 422 million people globally and is attributed directly to 1.5 million deaths each year, it is a serious public health issue [1]. It also places a significant burden on the healthcare systems of individual countries, given that the estimated global direct health cost in 2019 exceeded $760 million, indicating that it is one of the most expensive chronic metabolic conditions [2]. As type 2 diabetes mellitus (T2DM) accounts for 90% to 95% of all diagnosed diabetes cases [3], it should be the focus of preventative measures. Although T2DM is a multifactorial disease and its development is partly influenced by genetic heredity, personal lifestyle (such as diet and amount of exercise) plays an important role not only in its emergence but also in the affected individual’s ability to achieve adequate glycemic control [4]. However, a growing body of research (including the pioneering CDC Kaiser Permanente study) indicates that adverse childhood experiences (ACEs), i.e., adverse life events that occur before the age of 18 years, can increase the risk of the emergence of non-communicable diseases (NCDs), including T2DM, later in life [5].

ACEs can range from physical, emotional and sexual abuse to neglect resulting from household dysfunction caused by parental mental illness, substance abuse, incarceration, domestic violence and/or divorce [5]. In clinical and research settings, the presence of ACEs in an individual’s life history is typically established by administering a 10-item recall questionnaire [6]. According to a CDC report issued in 2022 [7], globally, around 17% of all adults surveyed regarding their history of ACEs reported experiencing at least four different types of ACE irrespective of sex and cultural setting. Nonetheless, ACEs are more prevalent in low- and middle-income countries like Bangladesh. Here an astonishing 99% [8] of surveyed children reported experiencing physical abuse, and 97% [9] reported suffering psychological abuse at home.

These statistics are alarming on their own, but given that ACEs have been demonstrated to increase the risk of a wide range of developmental difficulties, they warrant urgent action. According to the Biological Embedding of Childhood Adversity Model, childhood maltreatment and family dysfunction lead to impaired neurodevelopment, thus compromising healthy emotional, behavioral, and physical development. Consequently, ACEs significantly contribute to disease and mortality in adulthood [5, 10, 11]. This model explicitly proposes that childhood stress caused by poverty or maltreatment increases cell propensity for pro-inflammatory responses, resulting in exaggerated cytokine production prompted by stress. Persistent stress early in life also decreases sensitivity to inhibitory hormonal signals, as well as hormonal dysregulation, which typically manifests through risky behaviors (such as unhealthy lifestyle choices) while increasing the likelihood of NCDs such as diabetes mellitus in adulthood [12–14].

However, a positive association between ACEs and T2DM is confirmed in some studies [13, 14], while others suggest that no definitive link can be established [15]. Given that diabetes affects 10% of the Bangladeshi population, placing the country 8^th^ globally in terms of diabetes prevalence [16], the disparities in existing evidence concerning ACEs and T2DM demand further examination. In particular, it is crucial to identify factors contributing to this high disease burden beyond aging, obesity, and lack of exercise. Given the high incidence of ACEs in Bangladesh and the lack of studies linking the connection between ACEs to T2DM in adulthood life, this gap in the literature has prompted the current investigation.

## Materials and methods

### Study population

The study sample was recruited from Sirajdikhan (23°30’ and 23°41’ latitude and 90°14’ and 90°27’ longitude), a sub-district of Munshiganj located around 29 kilometers southeast of Dhaka, the capital city of Bangladesh during the period of 1^st^ March 2022 to 30^th^ June 2022. This subdistrict, predominantly agricultural area measuring around 180 km^2^ in size has been the subject of surveillance by the Department of Public Health and Informatics, Bangabandhu Sheikh Mujib Medical University (BSMMU) since 2015. Furthermore, a diabetic cohort has been maintained in a private healthcare facility for the last 10 years. Approximately 2,000 diabetic patients residing in Sirajdikhan area regularly visit this center to receive health care making it an ideal setting for the present investigation. All of these patients have a unique identification number, which served as the sampling frame for this study. Participants for T2DM group were randomly selected using this frame. To form an age-matched healthy control group (age range 30 to 65 years), adults from the neighboring households in the same community were invited to participate in the investigation. The prevalence of T2DM increases with age [17], so age-matched healthy controls were selected to improve statistical precision [18].

### Sample size

The sample size required for meeting the study objectives was calculated considering a two-sided confidence level (1 − alpha) of 95%, a minimum statistical power of 80%, an alpha level of 5%, one control matched to each case subject, and a hypothetical proportion of exposure in the control group of 15.0%. Given that, according to Lown et al. [19], the odds ratio (OR) of the association between exposure to 4+ ACEs and developing DM was 2.51 with a 95% confidence interval (CI) of 1.12 to 5.63, based on these values, the minimum sample size in this study was 137 for each group. Thus, finally, 137 diabetic patients and 134 healthy adults were recruited.

### Study variables

The study focused on Adverse Childhood Experiences (ACEs), utilizing the ACE questionnaire 10-item version (ACE-10), a self-reported retrospective questionnaire, to gather data on participant adversities before age 18 [20]. It has been proven culturally valid in Bangladesh [21] and is a well-validated tool globally, demonstrating good internal consistency with a Cronbach’s alpha of 0.70 [22]. The ACE questionnaire tool was translated in Bengali, the official and colloquial language of Bangladesh. The questionnaire investigates ten types of intrafamilial ACEs: psychological, physical, and sexual abuse; emotional and physical neglect; parental separation; violence against the mother; household substance abuse; household mental illness; and incarceration of any household member. When completing the questionnaire, respondents are required to provide "yes" or "no" answers, where an affirmative response to any question is coded as exposure to the relevant event and is assigned a value of one. Based on the number of types of ACEs, a cumulative ACE score is calculated by adding the number of “yes” answers. This results in a score between 0 and 10, which serves as a severity index indicating how many adversities someone experienced in their childhood [23].

### Confounding variables

As the study aim was to test for a link between ACEs and T2DM, information relevant for assessing T2DM severity and the level of glycemic control was obtained from all participants. Specifically, weight and height were measured to calculate body mass index (BMI), which was categorized according to Asian-pacific cutoff points ‘23 kg/m^2^’ to identify individuals in the “overweight and obese” category [24]. The weight was measured using the same digital scales, and height was measured using a standard tape. To ensure accuracy, participants were instructed to remove heavy clothing and footwear during both measurements. Likewise, in accordance with the World Health Organization (WHO) guidelines, blood pressure (BP) was measured three times at 5-minute intervals using a digital BP apparatus (Omran) while participants were in a sitting position with their right arm extended and slightly flexed [25]. The standard protocol was followed, and the average of the last two readings was retained for analyses. In addition, in line with the guidelines established by Bonita et al. [26] and Booth et al. [27], hypertension was defined as having a systolic blood pressure (SBP) equal to or greater than 140 mm of Hg and/or diastolic blood pressure (DBP) equal to or greater than 90 mm of Hg, or being treated for hypertension. For capturing health risks that could contribute to poor T2DM management, patients were asked to report smoking or consuming smokeless tobacco. For estimating stress levels the participants typically experience, the four-item Perceived Stress Scale-4 (PSS-4) was adopted [28]. This state measure was chosen due to its adequate reliability (with a Cronbach’s alpha coefficient of 0.60). When completing this instrument, participants were instructed to rate the frequency of stressful events in the past month on a scale from 0 (never) to 4 (very often) [29]. To account for the heredity of DM, the family history of DM was ascertained, and the pertinent socio-demographic characteristics were recorded, namely age, sex, marital status (married, widowed, divorced/separated, single/never married), level of education, employment status, and family structure (single or extended). Education levels were stratified as primary (grades I to V), secondary (grades VI to X), higher secondary and beyond (above grade X), and those lacking formal institutional education, categorized as non-institutional. Employment status was classified into unemployed, housewife, sedentary service (involving office duties without physical exertion), and active service (comprising physically demanding jobs such as day laborer, garment laborer, and farming).

### Biochemical measurements

After collecting the aforementioned data from the participants, we invited them to attend a private healthcare facility for biochemical blood tests that would provide a reliable indication of their overall health status and assess their glycemic control level. These tests included measuring their fasting and 2-hours after postprandial blood sugar levels, as well as their HbA1c and fasting lipid profile (i.e., total cholesterol, triglyceride, HDL, and LDL). For this purpose, a registered phlebotomist collected 8.0 ml of venous blood on two occasions, as required for further analyses. A participant was considered to have dyslipidemia if any of the following criteria were met: serum total cholesterol > 200 mg/dl, LDL-cholesterol ≥ 140 mg/dl, HDL-cholesterol < 40 mg/dl, triglycerides ≥ 150 mg/dl, and/or currently receiving treatment with lipid-lowering medications [30]. All blood samples were sent to the Department of Laboratory Medicine, BSMMU, within six hours of collection while maintaining a proper cold chain. Serum glucose was measured by the hexokinase method and the other tests (with the exception of LDL-cholesterol) were conducted by standard enzymatic assay using an automated biochemical analyzer (Beckman Coulter AU480, Tokyo, Japan). For determining LDL-cholesterol concentrations, the Friedewald formula was adopted [31]. The intra- and inter-assay coefficients of variation for the tests were within 2.5% and 4%, respectively.

### Ethical considerations

As data collection on childhood adversity is a sensitive issue, ethical and methodological challenges may arise, due to which such information is rarely elicited from patients or research participants in order to avoid re-traumatization. However, as the aim of the present study was to assess the association between ACEs and T2DM, evoking those potentially distressing memories was unavoidable. Thus, to reduce the discomfort caused to the participants, they were interviewed in private by trained interviewers after providing informed written consent. They were also informed of the study aims and were told that the information they divulged, would be used solely for research purposes. Likewise, they were assured that they could withdraw from the study and refuse to respond to any questions. Before the study, ethical permission was obtained from the Institutional Review Board of the University of Dhaka (Ref. No. 152/Biol. Scs. 2021). This study is part of a broader research work titled "Elucidating genetic and epigenetic mechanisms of type 2 diabetes mellitus pathogenesis".

### Statistical analyses

Descriptive analysis was performed on the sociodemographic data and other predictive factors, while rate and proportion were calculated for categorical variables. For continuous variables, mean (standard deviation, SD), median, maximum, and minimum were calculated. The normality of data distribution was assessed through the Shapiro-Wilk test, whereby a p-value < 5% was considered indicative of an asymmetric distribution. The unpaired t-test was performed to compare symmetrically distributed continuous variables, whereas asymmetrically distributed continuous variables were subjected to the Mann-Whitney U test. Moreover, Chi-squared statistics were calculated to compare categorical variables, while both univariable and multivariable binary logistic regression analyses were performed to determine presence of any significant associations between T2DM and, ACE and other independent variables. Here, T2DM was considered as outcome variable. Odds ratios (ORs) and 95% confidence intervals (CIs) were calculated, and a p-value < 0.05 was considered statistically significant. A window-based statistical software package, SPSS version 26, was used for all analyses.

## Results

### Socio-demographic, biochemical, and clinical characteristics

As shown in Table 1, the average age of the study participants in the T2DM and the control group was comparable (46.9 vs. 45.7 years, p >0.05), with the age range of 30 to 65 years for T2DM group and that of in control group 32 to 65 years. The mean (SD) duration of diabetes in the T2DM group was 6.95 (3.93) years. Females were predominant in both groups (69.3% vs. 61.2%, p < 0.05) and their mean (SD) BMI was comparable [T2DM:26.30 (3.97) vs. Controls:25.92 (4.11) kg/m^2^]. On the other hand, T2DM patients had significantly higher blood sugar levels, HbA1c values and serum triglyceride levels than healthy controls (p <0.0001). Finally, participants with T2DM had an average PSS-4 score of 8.6 out of 16, while healthy participants had an average score of 6.57 (p < 0.0001).

**Table 1:** Socio-demographic, anthropometric, laboratory, and behavioral characteristics of the study cohort (n = 271).

Characteristics	Healthy Control (n = 134)	T2DM (n = 137)	p-value
**Sex, n(%)**			
Male	52 (38.8)	42 (30.7)	0.159
Female	82 (61.2)	95 (69.3)	
**Age in years, mean (SD)**	45.66 (8.68)	46.89 (7.82)	0.220
**Education, n(%)**			
Non-institutional	28 (20.9)	22 (16.1)	0.731
Primary	23 (17.2)	22 (16.1)	
Secondary	64 (47.8)	71 (51.8)	
Higher secondary and above	19 (14.2)	22 (16.1)	
Occupation*, **n(%)**			
Sedentary service	30 (22.4)	28 (20.4)	
Active service	20 (14.9)	16 (11.7)	
Housewife	77 (57.5)	90 (65.7)	0.378
Unemployed	7 (5.2)	3 (2.2)	
**Smoking, n (%)**	20 (14.9)	16 (11.7)	0.198
**Smokeless tobacco, n (%)**	15 (9.2)	24 (17.5)	0.251
**Overweight and obese** **(BMI > 23 kg/m** ^**2**^ **)#, n (%)**	103 (76.9)	107 (78.1)	0.885
**Fasting blood sugar (mmol/L), median (IQR)**	5.3 (0.6)	9.7 (6.1)	<0.0001
**2-hr postprandial blood sugar (mmol/L), median (IQR)**	6.4 (1.3)	14.4 (8.8)	<0.0001
**HbA1c (%), median (IQR)**	5.6 (0.4)	8.7 (3.8)	<0.0001
**Serum lipid profile, mean (SD)**			
Total cholesterol (mg/dl)	189.47 (47.85)	191.85 (55.21)	0.705
Triglyceride (mg/dl)	154.95 (83.72)	234.93 (189.30)	<0.0001
HDL (mg/dl)	46.51 (12.63)	43.73 (12.15)	0.067
LDL (mg/dl)	109.07 (33.44)	102.59 (38.93)	0.144
**Perceived stress scale-4, median (IQR)**	6 (6)	9 (5)	<0.0001
**Adverse childhood experience (ACE), median (IQR)**	3 (3)	4 (2)	0.002
**ACE category, n (%)**			
Score < 4	77 (57.5)	51 (37.2)	0.001
Score ≥ 4	57 (42.5)	86 (62.8)	
**Hypertension, n (%)**	42 (31.3)	88 (64.2)	<0.0001
**Family history hypertension, n (%)**	89 (66.4)	101 (73.7)	0.232
**Dyslipidemia, n (%)**	82 (61.2)	123 (89.8)	<0.0001
**Family history diabetes, n (%)**	80 (59.7)	111 (81)	<0.0001

*Sedentary service includes office duties and other types of work that do not involve physical exertion; Active service includes physically demanding jobs, such as day laborer, garment laborer, and farming.

#23kg/m^2^ is the cut-off point of overweight for Asia-pacific population

Here, IQR-Interquartile range, Mann-Whitney U test done for p-value of median (IQR)

### Adverse childhood experiences (ACEs)

Approximately 98% of participants reported experiencing at least one ACE during their early life. The most frequently reported ACEs among both T2DM patients and healthy controls were physical neglect (92.7% and 94%, respectively, p < 0.05) and physical abuse (78.1% and 72.4%, respectively, p <0.05). Specifically, a significantly higher percentage of participants in the T2DM group (69.3%) reported having a mother who was treated violently compared to the healthy controls (55.2%). Within the T2DM group, 28.5% of participants reported experiencing sexual abuse, the majority of whom were female, whereas in the control group, this figure was 18.7% with a slightly lower female proportion. These disparities were further reflected in the mean total ACE scale scores which were 3.96 for T2DM versus 3.34 for controls, (p < 0.001). Additionally, two-thirds of T2DM patients and less than half of the healthy controls reported having experienced at least four ACEs (Table 1 and S1 Table).

### Association between ACE and T2DM

In the multivariable binary logistic regression model, after adjustment for confounding factors such as age, sex and significant factors from the unadjusted model, experiencing at least four ACEs in childhood was associated with a 2.24-fold greater risk of T2DM in later life (OR_adj_ = 2.24; 95% CI = 1.238–4.061, p < 0.01). The adjusted model also revealed that female sex (OR_adj_ = 1.93; 95% CI = 1.022–3.662), hypertension (OR_adj_ = 3.92; 95% CI = 2.119–7.256), dyslipidemia (OR_adj_ = 5.14; 95% CI = 2.418–10.927), family history of DM (OR_adj_ = 2.95; 95% CI = 1.536–5.650), and PSS-4 score above 3 (OR_adj_ = 1.16; 95% CI = 1.072–1.264) were significant factors in the association between T2DM and ACE, as shown in Table 2.

**Table 2:** Crude and adjusted association between adverse childhood experiences (ACEs) and type 2 diabetes mellitus (T2DM).

Factors	OR_unadj_ (95% CI)	p-value	OR_adj_ (95% CI)	p-value
ACE group				
Score ≥ 4 (score < 4 is the ref.)	2.28 (1.40–3.71)	0.001	2.24 (1.2–4.06)	0.008
Age	1.018 (0.989–1.048)	0.219	1.02 (0.98–1.05)	0.454
Female (male is the ref.)	1.43 (0.87–2.37)	0.160	1.93 (1.02–3.66)	0.043
Hypertension (“no” is the ref.)	3.93 (2.37–6.52)	<0.0001	3.92 (2.12–7.26)	<0.0001
Dyslipidemia (“no” is the ref.)	5.57 (2.90–10.70)	<0.0001	5.14 (2.42–10.93)	<0.0001
Family history of DM (“no” is the ref.)	1.88 (1.66–4.99)	<0.0001	2.95 (1.54–5.65)	0.001
PSS-4	1.17 (1.09–1.25)	<0.0001	1.16 (1.07–1.26)	<0.0001

*Notes*: Nagelkerke R^2^ = 0.396; here, adj–adjusted, OR–odds ratio, CI–confidence interval, ref–reference category, DM = diabetes mellitus, PSS = perceived stress scale, ACE = adverse childhood experience.

## Discussion

The study is considered pioneering because it examines the long-term effects of ACE on adult health, particularly on the development of DM, in Bangladesh. The findings suggest that ACEs are significantly associated with T2DM and may increase the risk of developing this condition. Specifically, our analyses revealed that a total ACE score exceeding 3 was associated with a 2.24-fold greater risk of T2DM in adulthood after accounting for other T2DM risk factors, including aging, female sex, hypertension, dyslipidemia, family history of diabetes, and current perceived stress.

Earlier studies have revealed an association between T2DM and ACEs, including the landmark study conducted by Felitti et al. [5], which found that individuals with a history of ACEs were at a higher risk of developing T2DM. These researchers developed an ACE scale ranging from 0 to 7 and determined that individuals scoring 4 or above were 1.6 times more likely to develop diabetes than those with lower scores. This finding has been replicated in subsequent research. For instance, in their prospective longitudinal study that followed participants from a British birth cohort up to 45 years of age, Thomas et al. [32] established that a history of ACEs was associated with a 20−50% increased risk of obesity, which in turn contributed to the development of T2DM (indicated by an HbA1c level greater than 6%) in adulthood.

In a population-based retrospective cohort study in the UK from 1995 to 2018, Chandan et al. [33] reported that individuals with a history of childhood maltreatment had a 71%, 42%, 113%, and 75% increased risk of subsequent cardiovascular disease, hypertension, T2DM, and all-cause mortality, respectively. After adjusting for key covariates, the analyses further revealed that individuals with a history of ACE faced an increased risk of T2DM (adjusted incidence rate ratio 2.13; 95% CI = 1.86–2.45) during the study period compared to those without history of ACEs.

The association between ACEs and the development of diabetes, along with other chronic physical conditions has been ascribed to diverse factors, including increased chronic stress that, if it progresses to toxic stress [34] over time, would predispose the affected individual to subclinical hypercortisolism early in life [35]. Such adverse experiences during critical development stages can compromise neurodevelopment, specially affecting hypothalamic−pituitary−adrenal (HPA) axis. This disruption leads to sustained increase in blood glucose, free fatty acid and triglyceride levels [36]. Kalmakis et al. [37] also pointed out that a history of childhood stress is significantly related to chronically low levels of cortisol. Furthermore, evidence indicates that ACEs serve as a persistent stimulus, causing an increase in the production of pro-inflammatory cytokines, including TNF-alpha and IL-6 [38] if adverse conditions persist. This results in a low-grade inflammatory state, which elevates the risk of developing insulin resistance [39].

In recent years, research on epigenetic changes has proliferated and the findings indicate that ACEs may trigger biological changes thereby increasing the likelihood of disease occurrence [40, 41]. Extant studies in this domain suggest that long-term changes in an organism’s developmental trajectory (denoted as developmental plasticity) may occur as a result of gene−environment interactions. These interactions result in alterations in gene expression through epigenetic mechanisms such as histone modifications and DNA methylation [42, 43]. These postulates were corroborated by an animal study conducted by Weaver et al. [44], which indicate that low levels of maternal care in rodents result in greater methylation of the promoter region of the hippocampal glucocorticoid receptor gene and alter the action of HPA axis. Moreover, Jiang et al. [45] have found that epigenetic silencing of genes in the stress pathway (the HPA axis) due to childhood trauma is implicated in the emergence of several chronic diseases, including T2DM.

When interpreting the findings reported here, several study limitations need to be noted, one of which stems from the retrospective data collection pertaining to ACEs. This strategy may have resulted in recall bias, even though traumatic experiences are usually well-remembered. Moreover, considering the sensitive nature of the questions, some participants may have underreported their traumatic experiences. We have made efforts to mitigate this issue by assuring the anonymity of the participants and maintaining strict confidentiality.

Given that diabetes is an incurable chronic disorder with numerous complications and a huge economic cost that significantly decreases patients’ quality of life and places a tremendous strain on their family, society, and country, effective diabetes care is crucial for delaying or reversing diabetes progression. Presently diabetes management focuses on lifestyle modifications and pharmacological treatment. However, as shown in this study, disease management strategies currently utilized should be supplemented by early prevention strategies. In particular, given the strong association between ACEs and T2DM in the Bangladeshi cohort examined in this study, the aim should be to reduce the direct and indirect effects of ACEs on further life trajectories, thereby preventing the onset of chronic diseases, including T2DM. Indeed, several authors view early detection of ACEs as a promising strategy to improve child well-being through legislation, health education, and evidence-based initiatives for families, children, and adolescents [46]. Thus, the main contribution of this investigation stems from evidence suggesting that ACE screening should be routinely performed in primary healthcare settings.

## Conclusion

The current investigation revealed that having a higher ACE score is significantly associated with the risk of developing T2DM. Therefore, as the T2DM prevalence is increasing globally, ACE screening should be conducted in primary care settings. However, further molecular epigenetic studies are needed to understand the link between ACEs and T2DM better and identify mechanisms that can be targeted in preventive strategies.

## Supporting information

S1 Table Prevalence of adverse childhood experiences (ACEs) among healthy controls and type 2 diabetes mellitus (T2DM) patients. (DOCX)

## References

[1] Diabetes, World Health Organization 2022, https://www.who.int›health-topics

[2] Williams R, Karuranga S, Malanda B, Saeedi P, Basit A, Besancon S, et al. Global and regional estimates and projections of diabetes-related health expenditure: Results from the International Diabetes Federation Diabetes Atlas. Diabetes Res Clin Prac. 2020; 162: 108072. doi: 10.1016/j.diabres.2020.108072 32061820

[3] Deshpande AD, Harris-Hayes M, Schootman M. Epidemiology of diabetes and diabetes-related complications. Phys Ther. 2008;88(11):1254–64. doi: 10.2522/ptj.20080020 18801858 PMC3870323

[4] Fletcher B, Gulanick M, Lamendola C. Risk factors for type 2 diabetes mellitus. J Cardiovas Nurs. 2002;16(2):17–23. 10.1097/00005082-200201000-00003 11800065

[5] Felitti VJ, Anda RF, Nordenberg D, Williamson DF, Spitz AM, Edwards V, et al. Relationship of childhood abuse and household dysfunction to many of the leading causes of death in adults: The adverse childhood experiences (ACE) study. Am J Prev Med. 1998;14(4):245–258. doi: 10.1016/S0749-3797(98)00017-8 9635069

[6] Zarse EM, Neff MR, Yoder R, Hulvershorn L, Chambers JE, Chambers RA. The adverse childhood experiences questionnaire: Two decades of research on childhood trauma as a primary cause of adult mental illness, addiction, and medical diseases. Cogent Med. 2019;6(1):1581447. doi: 10.1080/2331205X.2019.1581447

[7] CDC, 2022; Fast Facts: Preventing Adverse Childhood Experiences. Available from: **https://www.cdc.gov/violenceprevention/aces/fastfact.html**

[8] Haque MA, Janson S, Moniruzzaman S, Rahman AF, Islam SS, Mashreky SR, et al. Children’s exposure to physical abuse from a child perspective: A population-based study in rural Bangladesh. PLoS One. 2019;14(2):e0212428. doi: 10.1371/journal.pone.0212428 30779784 PMC6380542

[9] Haque MA, Moniruzzaman S, Janson S, Rahman AF, Mashreky SR, Eriksson UB. Children’s exposure to psychological abuse and neglect: A population based study in rural Bangladesh. Acta Paediatrica. 2021; 110(1): 257–264. doi: 10.1111/apa.15340 32368813

[10] Miller GE, Chen E, Parker KJ. Psychological stress in childhood and susceptibility to the chronic diseases of aging: moving toward a model of behavioral and biological mechanisms. Psychol Bull. 2011; 137(6):959–997. doi: 10.1037/a0024768 21787044 PMC3202072

[11] Berens AE, Jensen SK, Nelson CA. Biological embedding of childhood adversity: from physiological mechanisms to clinical implications. BMC Med. 2017;15(1):1–2. doi: 10.1186/s12916-017-0895-4 28724431 PMC5518144

[12] Almuneef M, Hollinshead D, Saleheen H, AlMadani S, Derkash B, AlBuhairan F, et al. Adverse childhood experiences and association with health, mental health, and risky behavior in the kingdom of Saudi Arabia. Child Abuse & Neglect. 2016 Oct 1;60:10–7. doi: 10.1016/j.chiabu.2016.09.003 27662614

[13] Hughes K, Bellis MA, Hardcastle KA, Sethi D, Butchart A, Mikton C, et al. The effect of multiple adverse childhood experiences on health: a systematic review and meta-analysis. Lancet Public Health. 2017;2(8):e356–66. doi: 10.1016/S2468-2667(17)30118-4 29253477

[14] Huang H, Yan P, Shan Z, Chen S, Li M, Luo C, et al. Adverse childhood experiences and risk of type 2 diabetes: A systematic review and meta-analysis. Metabolism. 2015;64(11):1408–18. doi: 10.1016/j.metabol.2015.08.019 26404480

[15] Subramaniam M, Abdin E, Vaingankar JA, Chang S, Sambasivam R, Jeyagurunathan A, et al. Association of adverse childhood experiences with diabetes in adulthood: results of a cross-sectional epidemiological survey in Singapore. BMJOpen. 2021;11(3):e045167. doi: 10.1136/bmjopen-2020-045167 33722874 PMC7959232

[16] Akhtar S, Nasir JA, Sarwar A, Nasr N, Javed A, Majeed R, et al. Prevalence of diabetes and pre-diabetes in Bangladesh: a systematic review and meta-analysis. BMJ Open. 2020;10(9):e036086. doi: 10.1136/bmjopen-2019-036086 32907898 PMC7482481

[17] Mordarska K, Godziejewska-Zawada M. Diabetes in the elderly. Menopause Review. 2017;16(2):38–43. doi: 10.5114/pm.2017.68589 28721127 PMC5509969

[18] Pearce N. Analysis of matched case-control studies. BMJ. 2016; 352: i969. doi: 10.1136/bmj.i969 26916049 PMC4770817

[19] Lown EA, Lui CK, Karriker-Jaffe K, Mulia N, Williams E, Ye Y, et al. Adverse childhood events and risk of diabetes onset in the 1979 National longitudinal survey of youth cohort. BMC Public Health. 2019;19:1–3. doi: 10.1186/s12889-019-7337-5 31351463 PMC6661082

[20] Anda R, Tietjen G, Schulman E, Felliti V, Croft J. Adverse childhood experiences and frequent headaches in adults. Headache: The Journal of Head and Face Pain. 2010;50(9):1473–1481. doi: 10.1111/j.l526-4610.2010.01756.x 20958295

[21] Haque MA, Tasnim A, Islam S, Towhid MI, Sultana S, Salwa M, et al. Mother’s adverse childhood experiences and elevated risk of neuro-developmental disorders in offspring: a case–control study. Adversity and Resilience Science. 2021;2(3):205–14. doi: 10.1007/s42844-021-00042-w

[22] Oláh B, Fekete Z, Kuritárné Szabó I, Kovács-Tóth B. Validity and reliability of the 10-Item Adverse Childhood Experiences Questionnaire (ACE-10) among adolescents in the child welfare system. Front Public Health. 2023;11:1258798. doi: 10.3389/fpubh.2023.1258798 38045975 PMC10691263

[23] CDC, about the CDC-Kaiser ACE study, 2021. Available from: https://www.cdc.gov/violenceprevention/aces/about.html

[24] Lim JU, Lee JH, Kim JS, Hwang YI, Kim TH, Lim SY, et al. Comparison of world health organization and asia-pacific body mass index classifications in COPD patients. Int J Chron Obstruct Pulmon. 2017;21:2465–2475. doi: 10.2147/COPD.S141295 28860741 PMC5571887

[25] WHO, 2012, WHO technical specifications for automated non-invasive blood pressure measuring devices with cuff. Available from: https://www.who.int›docs›searo›indonesia 10.1161/HYPERTENSIONAHA.120.16625 PMC7884242 33517681

[26] Bonita R, DeCourten M, Dwyer T, Jamrozik K, Winkelmann R. Surveillance of risk factors for noncommunicable disease: the WHO STEPwise approach [WHO document WHO/NMH/CCS/01.2002]. Geneva, Switzerland: World Health Organization; 2002.

[27] Booth GL, Kapral MK, Fung K, Tu JV. Relation between age and cardiovascular disease in men and women with diabetes compared with non-diabetic people: a population-based retrospective cohort study. Lancet. 2006;368(9529):29–36. doi: 10.1016/S0140-6736(06)68967-8 16815377

[28] Cohen S, Kamarck T, Mermelstein R. A global measure of perceived stress. Journal of Health and Social Behavior. 1983; 24:385–396. doi: 10.2307/2136404 6668417

[29] Ingram IV PB, Clarke E, Lichtenberg JW. Confirmatory factor analysis of the perceived stress Scale‐4 in a community sample. Stress and Health. 2016;32(2):173–6. doi: 10.1002/smi.2592 24995556

[30] Kinoshita M, Yokote K, Arai H, Iida M, Ishigaki Y, Ishibashi S, et al. Japan Atherosclerosis Society (JAS) guidelines for prevention of atherosclerotic cardiovascular diseases 2017. Journal of Atherosclerosis and Thrombosis. 2018;25(9):846–984. doi: 10.5551/jat.GL2017 30135334 PMC6143773

[31] Friedewald WT, Levy RI, Fredrickson DS. Estimation of the concentration of low-density lipoprotein cholesterol in plasma, without use of the preparative ultracentrifuge. Clinical Chemistry. 1972;18(6):499–502. 4337382

[32] Thomas C, Hyppönen E, Power C. Obesity and type 2 diabetes risk in mid adult life: the role of childhood adversity. Pediatrics. 2008;121(5):e1240–9. 18450866 10.1542/peds.2007-2403

[33] Chandan JS, Okoth K, Gokhale KM, Bandyopadhyay S, Taylor J, Nirantharakumar K. Increased cardiometabolic and mortality risk following childhood maltreatment in the United Kingdom. J Am Heart Assoc. 2020;9(10):e015855. doi: 10.1161/JAHA.119.015855 32410481 PMC7660837

[34] Shonkoff JP, Garner AS, Committee on Psychosocial Aspects of Child and Family Health, Committee on Early Childhood, Adoption, and Dependent Care, and Section on Developmental and Behavioral Pediatrics, et al. The lifelong effects of early childhood adversity and toxic stress. Pediatrics. 2012;129(1):e232–46. doi: 10.1542/peds.2011-2663 22201156

[35] Diz-Chaves Y, Gil-Lozano M, Toba L, Fandino J, Ogando H, Gonzalez-Matiac LC, et al. Stressing diabetes? The hidden links between insulinotropic peptides and the HPA axis. J Endocrinol. 2016;230(2):R77–94. doi: 10.1530/JOE-16-0118 27325244

[36] Dempster KS O’Leary DD, MacNeil AJ, Hodges GJ, Wade TJ. Linking the hemodynamic consequences of adverse childhood experiences to an altered HPA axis and acute stress response. Brain, Behavior, and Immunity. 2021;93:254–63. doi: 10.1016/j.bbi.2020.12.018 33358983

[37] Kalmakis KA, Meyer JS, Chiodo L, Leung K. Adverse childhood experiences and chronic hypothalamic–pituitary–adrenal activity. Stress. 2015;18(4):446–50. doi: 10.3109/10253890.2015.1023791 25783196

[38] Gill H, El-Halabi S, Majeed A, Gill B, Lui LM, Mansur RB, et al. The association between adverse childhood experiences and inflammation in patients with major depressive disorder: a systematic review. JAffect Disord. 2020;272:1–7. doi: 10.1016/j.jad.2020.03.145 32379599

[39] Chen L, Chen R, Wang H, Liang F. Mechanisms linking inflammation to insulin resistance. Int J Endocrinol. 2015. doi: 10.1155/2015/508409 26136779 PMC4468292

[40] Yang BZ, Zhang H, Ge W, Weder N, Douglas-Palumberi H, Perepletchikova F, et al. Child abuse and epigenetic mechanisms of disease risk. AmJ Prev Med. 2013;44(2):101–7. doi: 10.1016/j.amepre.2012.10.012 23332324 PMC3758252

[41] Palma-Gudiel H, Córdova-Palomera A, Leza JC, Fananas L. Glucocorticoid receptor gene (NR3C1) methylation processes as mediators of early adversity in stress-related disorders causality: a critical review. Neurosci Biobehav Rev. 2015;55:520–35. doi: 10.1016/j.neubiorev.2015.05.016 26073068

[42] Kaufman J, Montalvo-Ortiz JL, Holbrook H, O’Loughlin K, Orr C, Kearney C, et al. Adverse Childhood Experiences, Epigenetic Measures, and Obesity in Youth. J Pediatr. 2018;202:150–156.e3. doi: 10.1016/j.jpeds.2018.06.051 30177354 PMC6513669

[43] Hochberg ZE. Developmental plasticity in child growth and maturation. Front Eendocrinol. 2011;2:41. doi: 10.3389/fendo.2011.00041 22666215 PMC3364458

[44] Weaver IC, Meaney MJ, Szyf M. Maternal care effects on the hippocampal transcriptome and anxiety-mediated behaviors in the offspring that are reversible in adulthood. Proceedings of the National Academy of Sciences. 2006;103(9):3480–5. doi: 10.1073/pnas.0507526103 16484373 PMC1413873

[45] Jiang S, Postovit L, Cattaneo A, Binder EB, Aitchison KJ. Epigenetic modifications in stress response genes associated with childhood trauma. Front Psychiatry. 2019;10:808. doi: 10.3389/fpsyt.2019.00808 31780969 PMC6857662

[46] Gordon JB, Nemeroff CB, Felitti V. Screening for adverse childhood experiences. JAMA. 2020;324(17):1788–89. doi: 10.1001/jama.2020.16449 33141202

