# Correction: Association between adverse childhood experiences and type 2 diabetes mellitus in later life: A case-control study

**DOI:** 10.1371/journal.pgph.0006352

**Published:** 2026-04-21

**Authors:** Nilima Barman, Abul B. M. M. K. Islam, M. Atiqul Haque

In the Socio-demographic, biochemical, and clinical characteristics subsection of the Results, there is an error in the third sentence of the paragraph. The correct sentence is: Females were predominant in both groups (69.3% vs. 61.2%) and their mean (SD) BMI was comparable [T2DM:26.30 (3.97) vs. Controls:25.92 (4.11) kg/m2].

In the Adverse childhood experiences (ACEs) subsection of the Results, there is an error in the second sentence of the paragraph. The correct sentence is: The most frequently reported ACEs among both T2DM patients and healthy controls were physical neglect (92.7% and 94%, respectively) and physical abuse (78.1% and 72.4%, respectively).

S1 Table contained a typographical error in the row for Physical abuse. Please view the correct S1 Table below.

## Supporting information

S1 Table Prevalence of adverse childhood experiences (ACEs) among healthy controls and type 2 diabetes mellitus (T2DM) patients. (DOCX)
