# Supplementary material for: Correction: Association between adverse childhood experiences and type 2 diabetes mellitus in later life: A case-control study
Source: PLOS Glob Public Health. 2026 Apr 21;6(4):e0006352. doi: 10.1371/journal.pgph.0006352 (PMC13098971; doi:10.1371/journal.pgph.0006352)
Supplement: S1 Table — (DOCX) [file pgph.0006352.s001.docx]

**Supplementary Table ST1:** Prevalence of adverse childhood experiences (ACEs) among healthy controls and type 2 diabetes mellitus (T2DM) patients

| **Characteristics** | **Healthy Control (n = 134)** | **T2DM**  **(n = 137)** | **p-value** |
| --- | --- | --- | --- |
| **10 items ACEs, n(%)** |  |  |  |
| Physical abuse | 97 (72.4) | 107 (78.1) | 0.342 |
| Emotional abuse | 41 (30.6) | 58 (42.3) | 0.06 |
| Sexual abuse | 25 (18.7) | 39 (28.5) | 0.079 |
| Physical neglect | 126 (94) | 127 (92.7) | 0.845 |
| Emotional neglect | 44 (32.8) | 62 (45.3) | 0.049 |
| Parental separation | 6 (4.5) | 8(5.8) | 0.817 |
| Mother treated violently | 74 (55.2) | 95(69.3) | 0.023 |
| Substance abuse | 13 (9.7) | 17 (12.4) | 0.605 |
| Mental illness | 15 (11.2) | 17 (12.4) | 0.903 |
| Incarceration | 6 (4.5) | 13 (9.5) | 0.168 |
| **In 3 sections** |  |  |  |
| Abuse, mean (SD) | 1.22 (0.88) | 1.49 (0.92) | 0.013 |
| Neglect, mean (SD) | 1.27 (0.56) | 1.38 (0.61) | 0.121 |
| Household Dysfunction, mean (SD) | 0.85 (0.85) | 1.09 (0.83) | 0.018 |
